# Deep vein thrombosis in patients with stroke or transient ischemic attack presenting with patent foramen ovale: a retrospective observational study

**DOI:** 10.1186/s12883-024-03802-0

**Published:** 2024-08-26

**Authors:** Charlotte Huber, Stephan Stöbe, Andreas Hagendorff, Katja Sibylle Mühlberg, Karl-Titus Hoffmann, Berend Isermann, Rolf Wachter, Nikolaus von Dercks, Richard Schmidt, Johann Otto Pelz, Dominik Michalski

**Affiliations:** 1 Department of Neurology, University of Leipzig, Liebigstr. 20, 04103 Leipzig, Germany; 2 Department of Cardiology, University of Leipzig, Liebigstr. 20, 04103 Leipzig, Germany; 3 Department of Angiology, University of Leipzig, Liebigstr. 20, 04103 Leipzig, Germany; 4 Department of Neuroradiology, University of Leipzig, Liebigstr. 20, 04103 Leipzig, Germany; 5 Department of Laboratory Medicine, Clinical Chemistry and Molecular Diagnostics, University of Leipzig, Paul-List-Str. 13-15, 04103 Leipzig, Germany; 6 Department of Medical Management, University of Leipzig, Liebigstr. 18, 04103 Leipzig, Germany

**Keywords:** Deep vein thrombosis, Patent foramen ovale, Ischemic stroke, Transient ischemic attack

## Abstract

**Objective:**

Deep vein thrombosis (DVT) is discussed as a source of embolism for cerebral ischemia in the presence of patent foramen ovale (PFO). However, previous studies reported varying rates of DVT in stroke patients, and recommendations for screening are lacking. This study aimed to characterize patients with stroke or transient ischemic attack (TIA) and concomitant PFO and explore the rate of DVT and associated parameters.

**Methods:**

Medical records were screened for patients with stroke or TIA and echocardiographic evidence of PFO. Concomitant DVT was identified according to compression ultrasonography of the lower limbs. A variety of demographic, clinical, and laboratory parameters, the RoPE and Wells scores were compared between patients with and without DVT.

**Results:**

Three-hundred-thirty-nine patients (mean age 61.2 ± 15.4 years, 61.1% male) with stroke or TIA and PFO, treated between 01/2015 and 12/2020, were identified. Stroke and TIA patients did not differ for demographic and vascular risk factors. DVT was found in 17 cases out of 217 (7.8%) with compression ultrasonography. DVT was associated with a history of DVT, cancer, previous immobilization, calf compression pain, calf circumference difference, and a few laboratory abnormalities, e.g., increased D-dimer. A multivariate regression model with stepwise backward selection identified the Wells score (odds ratio 35.46, 95%-confidence interval 4.71–519.92) as a significant predictor for DVT.

**Conclusion:**

DVT is present in a relevant proportion of patients with cerebral ischemia and PFO, which needs to be considered for the individual diagnostic workup. The Wells score seems suitable for guiding additional examinations, i.e., compression ultrasonography.

## Introduction

Autoptic studies have indicated patent foramen ovale (PFO) in about 25% of the general population [1]. Among patients with ischemic stroke considered as a cryptogenic event, the probability of presenting a PFO was found to be three times higher as compared to different controls [1, 2]. Randomized controlled trials have investigated the closure of high-risk PFO in stroke patients and observed a decreased rate of recurrent events compared to medical treatment alone, which included different drugs affecting the coagulation system [3–5]. This led to the recommendation to consider PFO closure in selected stroke patients, including those with an age < 60 years, a high-risk PFO, and a lack of concurrent etiologic factors [6–8].

Despite the progress in handling stroke patients with cryptogenic stroke and coincident PFO, the underlying mechanism of PFO-associated stroke is still a matter of debate, and thus the individual diagnostic workup is rather challenging. A more recent perspective considers the PFO channel itself as the source of embolic events, while the traditional concept of paradoxical embolism comprised a deep vein thrombosis (DVT) as source of an embolus passing from the venous to the arterial system through the PFO [9, 10]. The concept of stroke due to paradoxical embolism was first described in 1877 [11], followed by a few case reports [12]. Remarkably, earlier studies yielded highly varying rates of DVT in stroke patients with PFO, ranging from 7 to 27% [13]. As the presence of a DVT was not an inclusion criterion of the randomized controlled trials showing beneficial effects of PFO closure [3–5], the discussion regarding causal relationships is still ongoing. Further, these trials excluded patients presenting with transient-ischemic attack (TIA), even though this group is at considerable risk for secondary events [14], which may be even higher in the presence of a PFO.

On the individual level, knowledge of a DVT in the setting of stroke and the presence of PFO seems to be important: First, to determine individual stroke etiology as best as possible, which is essential for secondary prevention, and second, to identify conditions that entail, at least for a certain period, an anticoagulation independent of the cerebral event. For reliable detection of DVT, ultrasonography has proven beneficial in neurological diseases [15], but its time-consuming aspect and a limited availability might inhibit systematic screenings. So far, there is no clear recommendation for the diagnostic workup for stroke patients presenting with a PFO, especially regarding a potential DVT [6, 16]. Uncertainties further exist regarding specific groups of patients, particularly those with TIA and an age older than 60 [10].

Therefore, this study aimed to characterize patients with stroke or TIA and concomitant PFO and explore the rate of DVT and associated parameters, which might help to guide the individualized diagnostic workup, particularly regarding additional examinations, i.e., ultrasonography.

## Methods

### Study design

For this retrospective, non-interventional study, hospital-based medical records of all stroke and TIA patients treated at the Department of Neurology of the University of Leipzig between January 2015 and December 2020 were screened for the International Statistical Classification of Diseases and Related Health Problems (ICD)-10 code I63.* (ischemic stroke) or G45.* (TIA) in conjunction with Q21.1 (PFO). Data obtained during the hospital stay were extracted from the electronic data storage systems.

The study was approved by the ethics committee of the Medical Faculty at the University of Leipzig (reference number 269/21-ek/) and performed in compliance with the ethical standards laid down in the 1964 Declaration of Helsinki and its later amendments. Further, the study was registered in “Deutsches Register klinischer Studien” (DRKS, reference number DRKS00025998). This study is reported according to STROBE guidelines [17].

### Patients, evaluation of PFO and DVT

Patients were included if they had an age of at least 18 years, a stroke or TIA, and evidence of PFO. According to current conventions, stroke was defined as focal neurological symptom presenting for more than 24 h or evidence of an ischemic lesion on cerebral imaging [i.e., magnetic resonance imaging (MRI) or computed tomography (CT)], while TIA was defined as a transient focal neurological symptom without evidence of infarction on cerebral imaging [18]. For TIA, at least one of the following symptoms had to be presented: aphasia, dysarthria, facial paresis, sensory or motor impairment of at least one extremity. Cases with uncertain TIA as for example possible peripheral-vestibular disorders (e.g., transient dizziness), migraine, and psychological disorders were excluded.

The presence of a PFO was evaluated by transthoracic and transesophageal echocardiography during routine examination, performed by a cardiologist and documented in medical records. In cases of uncertainty, the original echocardiographic images were re-evaluated by a cardiologist. Echocardiographic examinations were performed by Vivid 7, Vivid E9, or Vivid E95 ultrasound system with a M5-S or a 4Vc phased array probe (GE Healthcare Ultrasound Germany, Solingen/München, Germany), usually including the application of a contrast agent (Gelafusal 4%, Serumwerk Bernburg AG, Bernburg, Germany). Cases presenting pathologies not following the strict definition of a PFO, i.e., atrial septal defects (ASD) II, were excluded.

Regarding DVT, information was also taken from medical records describing the findings of a compression ultrasonography of the lower limbs, which was conducted by an angiologist. Ultrasound was carried out with commercial devices, e.g., LOGIQ E9, and Vivid E9 XDclear (GE Healthcare Germany, Solingen/München, Germany). Superficial vein thrombosis or muscle vein thrombosis was not rated as DVT.

### Included data

In addition to the presence of a PFO and DVT, the following patients’ characteristics were extracted from medical records: Demographic factors (age and sex), individual cardiovascular risk factors (arterial hypertension, hyper-/dyslipidemia, diabetes mellitus, nicotine consumption, atrial fibrillation), scores describing the previous general condition and the short-term functional outcome, i.e., modified Rankin Scale (mRS) [19] prior to the event (pre-mRS) and at hospital discharge, and National Institutes of Health Stroke Scale (NIHSS) [20] at admission and at discharge, existing medications affecting the coagulation system, and whether intravenous thrombolysis or endovascular therapy, i.e., mechanical recanalization, had been performed. Stroke etiology was classified according to the Trial of Org 10,172 in Acute Stroke Treatment (TOAST) criteria [21], added by the type “arterio-arterial embolism” to better describe the situation of a likely embolic event due to atherosclerosis in terms of plaque formation, usually located at the carotid arteries but not fulfilling the criteria of a stenosis with at least 50% or an occlusion.

Among well-established scores describing the risk for a DVT and the probability for a causal relationship between PFO and stroke, the Wells score [22] in its simplified version with one point for each criterion and the Risk of Paradoxical Embolism (RoPE) score [23] were used. Information on previous DVT, immobilization in the last 30 days before the qualifying cerebral event, current or past cancer, and current pulmonary embolism were included to assess the individual risk of thrombosis.

Clinical characteristics were calf compression pain and calf circumference difference. Laboratory parameters included, if available, D-dimer, C-reactive protein (CRP), leukocyte count, genetic thrombophilia (heterozygosity or homozygosity for prothrombin or factor V Leiden mutation), lupus anticoagulans-specific activated partial thromboplastin time (LA-specific aPTT), protein C, protein S, antinuclear antibodies (ANA), antibodies against beta-2-glycoprotein, cardiolipin, double strand DNA (dsDNA), nucleosomes, and histones were considered.

### Statistical analyses

Analyses were performed with SPSS software package version 29.0 (IBM corp., Armonk, NY, USA) and R Statistical Software [24] with R Studio [25]. Considering the sample size, non-parametric testing was applied for testing statistical significance between groups, including Mann-Whitney-U Test and Chi-square test or Fisher’s exact test. If suitable, odds ratios (OR) and 95% confidence intervals (CI) were calculated to describe the probability for the existence of a DVT under specific conditions. Further, a multivariate logistic regression model was built with stepwise backward selection using *p*-value and Akaike Information Criterion. Generally, a *p*-value of < 0.05 was considered statistically significant.

## Results

Of 388 patients identified, cases with stroke mimics, uncertain TIA, and echocardiographic findings indicating other (atrial) septal defects than typical PFO were excluded. Consequently, a total of 339 patients with PFO were included in the study, while subgroups of stroke and TIA patients comprised of 294 and 45 patients.

### Characterization of patients with stroke or TIA and concomitant PFO

Patients’ characteristics of the overall cohort and with reference to the subgroups stroke and TIA are shown in Table 1. Thereby, patients with cerebral ischemia and evidence for PFO exhibited a mean age of about 61 years, were more often male (61.1%), and had higher rates of arterial hypertension and hyperlipidemia. Most patients were classified to “arterio-arterial embolism” etiology, followed by “undetermined/competing” etiology.

**Table 1:** Patients’ characteristics

	Overall study group (*n* = 339)	Stroke (*n* = 294)	TIA (*n* = 45)	Statistical significance
Age, years (min-max, M (SD))	18–91, 61.2 (15.43)	18–91, 60.96 (15.61)	30–84, 62.73 (13.89)	n.s.
Sex (n (%))	Male 207 (61.1) Female 132 (38.9)	Male 183 (62.2) Female 111 (37.8)	Male 24 (53.3) Female 21 (46.7)	n.s.
Arterial hypertension (n (%))	245 (72.3)	213 (72.4)	32 (71.1)	n.s.
Diabetes mellitus (n (%))	87 (25.7)	78 (26.5)	9 (20.0)	n.s.
Hyperlipidemia (n (%))	219 (64.6)	191 (65.0)	28 (62.2)	n.s.
Nicotine consumption (n (%))	133 (39.2)	121 (41.2)	12 (26.7)	n.s.
Atrial fibrillation (n (%))	14 (4.1)	13 (4.4)	1 (2.2)	n.s.
Etiology (n (%))	Large-artery atherosclerosis 10 (2.9) Arterio-arterial embolism 126 (37.2) Cardioembolism 21 (6.2) Small-vessel occlusion 26 (7.7) Other 71 (21) Undetermined/competing 85 (25.1)	Large-artery atherosclerosis 8 (2.7) Arterio-arterial embolism 108 (36.7) Cardioembolism 20 (6.8) Small-vessel occlusion 24 (8.2) Other 65 (22.1) Undetermined/competing 69 (23.5)	Large-artery atherosclerosis 2 (4.4) Arterio-arterial embolism 18 (40.0) Cardioembolism 1 (2.2) Small-vessel occlusion 2 (4.4) Other 6 (13.3) Undetermined/competing 16 (35.6)	n.s.
Intravenous thrombolysis (n (%))	63 (18.6)	63 (21.4)	-	
Mechanical recanalization (n (%))	24 (7.1)	24 (8.2)	-	
Pre-mRS (M (SD))	0.21 (0.73)	0.24 (0.78)	0 (0.0)	*
NIHSS at admission (M (SD))	3.99 (6.29)	4.56 (6.57)	0.31 (0.63)	***
NIHSS at discharge (M (SD))	2.09 (5.49)	2.41 (5.84)	0 (0.0)	***
mRS at discharge (M (SD))	1.10 (1.42)	1.27 (1.45)	0 (0.0)	***
Medications affecting the coagulation system (n (%))	AP 80 (23.6) VKA 2 (0.6) NOAC 3 (0.9) LMWH/Heparin 2 (0.6) none 252 (74.3)	AP 69 (23.5) VKA 2 (0.7) NOAC 3 (1.0) LMWH/Heparin 2 (0.7) none 218 (74.1)	AP 11 (24.4) VKA 0 (0.0) NOAC 0 (0.0) LMWH/Heparin 0 (0.0) none 34 (75.6)	n.s.
TEE in addition to TTE (n (%))	333 (98.2)	288 (98.0)	45 (100.0)	n.s.
Compression ultrasonography of lower limbs (n (%))	217 (64.0)	196 (66.7)	21 (46.7)	*

Legend: TIA: Transient ischemic attack; M: Mean; SD: standard deviation; mRS: modified Rankin Scale; NIHSS: National Institutes of Health Stroke Scale; TTE: transthoracic echocardiography; TEE: transesophageal echocardiography; AP: antiplatelet agents; VKA: vitamin k antagonist oral anticoagulant; NOAC: non-vitamin K antagonist oral anticoagulant; n.s.: non-significant; *: *p* < 0.05, ***: *p* < 0.001

Patients with stroke or TIA did not differ significantly regarding sociodemographic factors, i.e., age and sex, and vascular risk factors, i.e., arterial hypertension, diabetes, hyperlipidemia, and atrial fibrillation (*p*-values 0.254–0.852). Compared to TIA patients, a numerically increased proportion of stroke patients presented with nicotine consumption (*p* = 0.064). Stroke patients exhibited an increased NIHSS at hospital admission (*p* < 0.001) and an increased pre-mRS (*p* < 0.001) compared to TIA patients, indicating more severe clinical symptoms at the time of cerebral ischemia and a poorer condition before the event. As expected, the stroke and TIA group differed regarding the short-term outcome: As patients with TIA naturally presented no impairment at hospital discharge, the stroke cohort showed minor neurological deficits and a relatively low disability indicated by low levels of NIHSS (*p* < 0.001) and mRS (*p* < 0.001). Regarding the etiology of cerebral ischemia, stroke and TIA patients did not differ significantly with reference to the used adapted TOAST criteria (*p* = 0.284). “Undetermined/competing” etiology was numerically more often in TIA patients, while “cardioembolism” was numerically more often in stroke patients. Regarding the pre-existing medical treatment, stroke and TIA patients did not differ significantly (*p* = 0.895), while most patients had no coagulation-modifying medication before admission.

As all Patients included in this study presented a PFO, whose detection is naturally easier on transesophageal echocardiography (TEE), a high proportion of stroke (98%) and TIA (100%) patients had this diagnostic investigation done during hospital stay.

### Rate of DVT

Compression ultrasonography of the lower limbs was conducted in 64% of the overall study group and a relatively wide temporal range of 1 to 15 days from hospital admission. Ultrasound examination was done significantly more often in stroke (66.7%) than in TIA (46.7%) patients (*p* = 0.016). In the overall study group comprising stroke and TIA patients with PFO, DVT was detected in 17 out of 339 cases (5%). For further calculations, only patients who underwent compression ultrasonography were considered. In those 217 patients, DVT was detected in 17 cases (7.8%). Remarkably, DVT was only found in stroke patients covering a group of 196 cases (8.8%), while compression ultrasonography performed in 21 TIA patients with PFO did not show evidence of DVT (0%). Despite this numerical difference in the rate of DVT among stroke and TIA patients examined with compression ultrasonography, statistical significance was not reached (*p* = 0.169). Regarding the dimension and location of DVT, of the 17 patients with DVT, 10 (58.8%) had a 1-level thrombosis, and 7 (41.2%) had a 2-level thrombosis. There were no 3- or 4-level thromboses. Of the 1-level thromboses, 6 (60%) were localized in the lower leg, 1 (10%) in the popliteal region, 2 (20%) in the thigh region, and 1 (10%) in the pelvic area. Regarding the 2-level thromboses, 4 (57.1%) were located distally in the lower leg and popliteal region and 3 (42.9%) in the thigh and popliteal or pelvic region. Regardless of the level, 10 of the 17 (58.8%) thromboses were distal and 7 (41.2%) proximal.

### Parameters associated with DVT

To explore parameters that may help to identify DVT in patients with PFO, the 17 cases with diagnosed DVT were compared with 200 cases without evidence for DVT by compression ultrasonography.

**Fig. 1:**
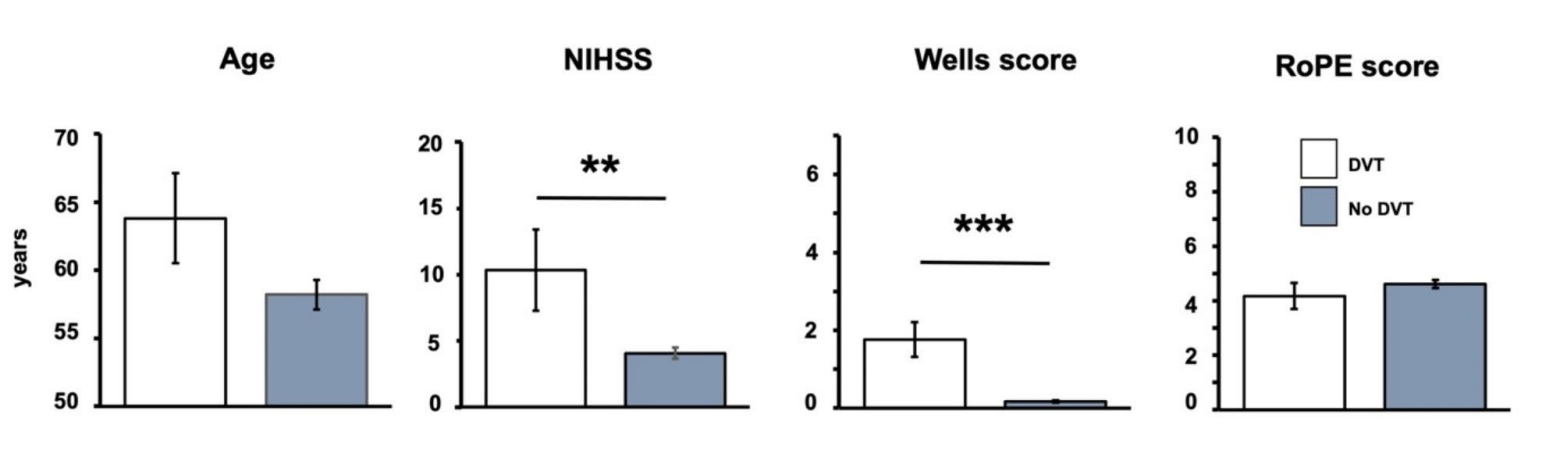
Comparison of age, National institutes of Health Stroke Scale (NIHSS) at hospital admission, Wells score, and RoPE score in stroke and TIA patients with and without deep vein thrombosis (DVT). Bars indicate mean values, added lines indicate standard error. **: *p* < 0.01, ***: *p* < 0.001. Number of patients in subgroup analyses (DVT/no DVT): age *n* = 17/*n* = 200, NIHSS *n* = 17/*n* = 200, Wells score *n* = 17/*n* = 200, and RoPE score *n* = 17/*n* = 200

Regarding demographic factors, patients with DVT were numerically older than those without (Fig. 1), failing statistical significance (*p* = 0.146). Further, sex did not differ significantly between patients with and without DVT (*p* = 0.226). Among clinical characteristics, patients with DVT exhibited a more severe neurological deficit as indicated by an increased NIHSS at hospital admission compared to those without DVT (*p* = 0.006). Regarding preexisting medications, patients with DVT compared to those without DVT did not differ concerning the existence of any medication affecting the coagulation system (17.6 vs. 25.5%; *p* = 0.5723).

Concerning already established approaches describing the risk for DVT (Fig. 1), an increased Wells score was found in stroke patients with PFO and concomitant DVT when compared to those without DVT (*p* < 0.001). Remarkably, a one-point increase in Wells score elevated the risk of DVT by 395% (OR 4.95, 95%-CI 2.73–10.52). Concerning the RoPE score, only 2 of the 17 patients (11.8%) with evidence of DVT had a score of 7 or higher, while 15 of the 17 patients (88.2%) had a score below 7. In the study group that is unselected for the cause of the ischemic event, the RoPE score did not differ significantly between patients with and without DVT (*p* = 0.360). Regarding anamnestic information covering the individual risk of thrombosis, patients with DVT more frequently had any type of cancer (23.5 vs. 9.5%; *p* < 0.001; OR 2.93, 95%-CI 0.77–9.28) and a history of DVT in the past (23.5 vs. 3.0%; *p* < 0.001; OR 9.95, 95%-CI 2.31–39.52). Also, patients with current DVT more often provided a coincident pulmonary artery embolism when compared to those without DVT (17.6 vs. 1% *p* < 0.001; OR 21.21, 95%-CI 3.27–171.52). If patients were immobilized in the last 30 days before the cerebrovascular event, DVT was detected more often than without prior immobilization (11.8 vs. 3.5%; *p* < 0.001; OR 10.36, 95%-CI 3.07–33.73).

In clinical assessments, patients with DVT compared to those without DVT more often presented calf compression pain (11.8 vs. 0.0%; *p* = 0.006) and calf circumference difference (23.5 vs. 0.01%; *p* < 0.001; OR 61.23, 95%-CI 8.35–1245.54).

**Fig. 2:**
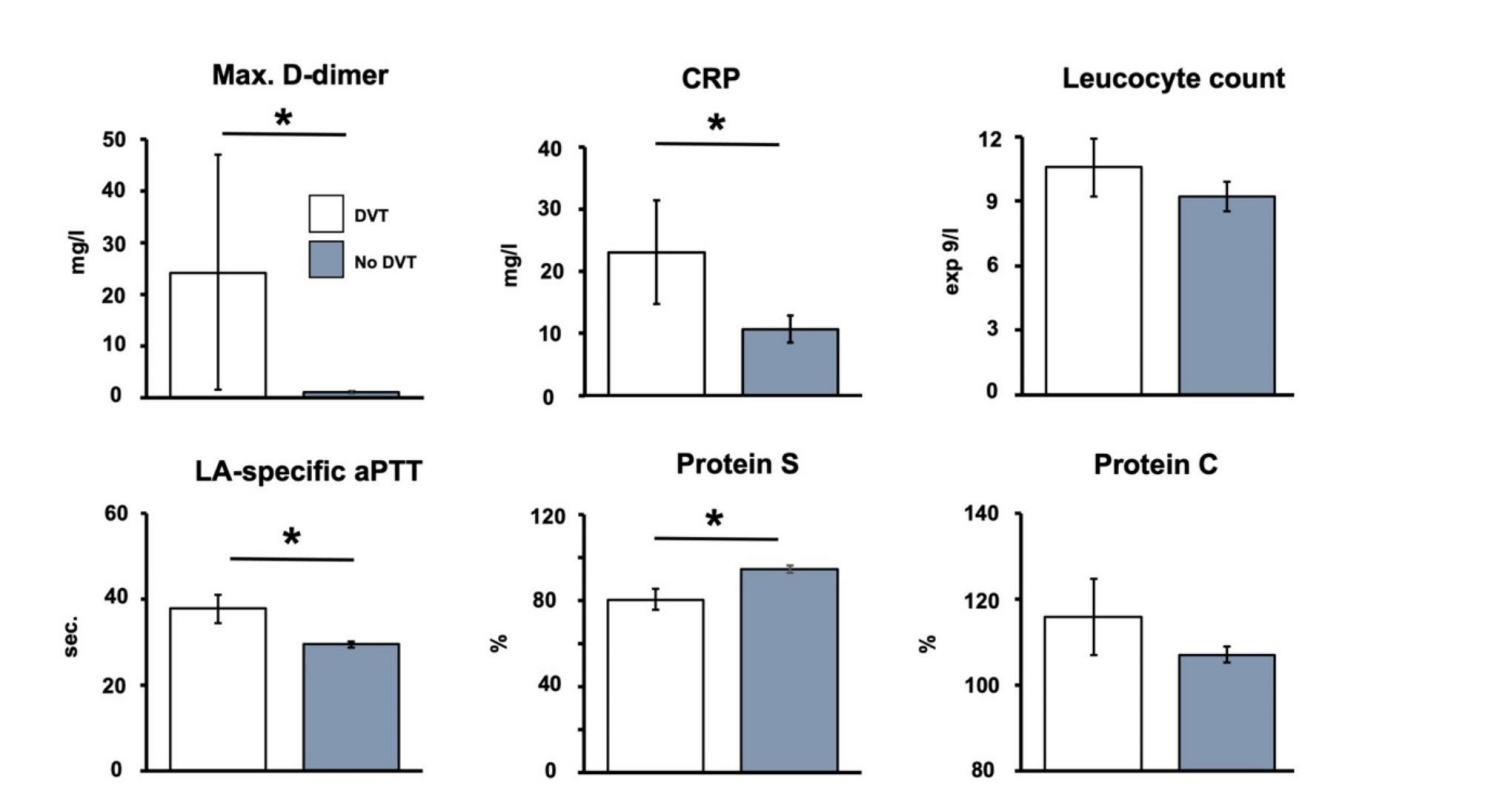
Comparison of selected laboratory parameters in stroke and TIA patients with and without deep vein thrombosis (DVT). Bars indicate mean values, added lines indicate standard error. *: *p* < 0.05. CRP: C-reactive protein, LA-specific aPTT: lupus anticoagulant-specific activated partial thromboplastin time. Number of patients in subgroup analyses (DVT/no DVT): max. D-dimer *n* = 3/*n* = 52, CRP *n* = 17/*n* = 200, leucocyte count *n* = 17/*n* = 200, LA-specific aPTT *n* = 12/*n* = 117, protein S *n* = 11/*n* = 113, and protein C *n* = 11/*n* = 114

Regarding laboratory parameters (Fig. 2), stroke patients with evidence of DVT, compared to those without, presented increased levels of D-dimer (*p* = 0.01), CRP (*p* = 0.038; OR 3.41, 95%-CI 1.25–9.81 for cut-off at 5 mg/L), and LA-specific aPTT (*p* = 0.032; OR 4.85, 95%-CI 1.89–17.06 for > 35 s.), while protein S was decreased (*p* = 0.015; OR 4.40, 95%-CI 1.04–16.76 for < 74%). However, patients with DVT were characterized by numerically increased leucocyte counts and levels of protein C without statistical significance (*p* = 0.249; *p* = 0.367). Remarkably, genetic thrombophilia was more often detected in patients with DVT as compared to those without DVT (42.9 vs. 7.6%; *p* = 0.010; OR 9.15, 95%-CI 1.48–55.02). Among antibodies, patients with DVT and those without DVT did not differ for ANA (*p* = 0.916), and antibodies directed against dsDNA (*p* = 0.139), nucleosomes (*p* = 0.213), histones (*p* = 0.058), beta-2-glycoprotein IgG (*p* = 0.572), beta-2-glycoprotein IgM (*p* = 0.614), cardiolipin IgG (*p* = 0.837), and cardiolipin IgM (*p* = 0.884).

A multivariate logistic regression model with DVT as the response variable and all formerly mentioned significantly differing parameters as predictors was applied, but the full model did not yield significant results. Remarkably, a stepwise backward selection model revealed the Wells score as a significant predictor for DVT in patients with cerebral ischemia and concomitant PFO (adjusted OR 35.46, 95%-CI 4.71–519.92).

## Discussion

This study aimed to characterize patients with stroke or TIA and concomitant PFO and also explore the rate of DVT and associated parameters, which might help to guide additional examinations, i.e., ultrasonography. Data from standard care of patients with cerebral ischemia and echocardiographically confirmed PFO were used, which might help to overcome limitations from earlier studies on highly selected populations.

With about 61 years of age, patients in this study were older compared to earlier investigations focusing on PFO closure, with about 43 and 45 years, respectively [3, 4]. Also, in studies primarily focusing on diagnostic proceedings for thrombosis in conjunction with PFO, patients were typically younger with about 47 and 57 years, respectively [26, 27]. The unselected nature of the cohort underlying this study is further emphasized by the finding that most events were etiologically classified to “arterio-arterial embolism”, while previous investigations, e.g., a PFO closure trial [3], excluded patients with causes other than the PFO, and non-interventional studies often focused on cryptogenic stroke [26]. Regarding subgroups stroke and TIA, both with concomitant PFO, the present study did not provide significant differences among age, sex, and typical risk factors. Following local standards, more than half of patients underwent compression sonography of the lower limbs, which was, however, more often performed after stroke when compared to TIA.

Focusing on patients with PFO and performed compression sonography of the lower limbs, the DVT rate in this study (7.8%) is in good accordance with earlier investigations. For example, DVT of the lower limbs was found in 7.1% of 131 patients with ischemic stroke or TIA with concomitant PFO [27]. Also, DVT was found in 7% of 293 patients with ischemic stroke and concomitant PFO [28]. In addition, DVT was detected in 8.7% of 323 patients in a study that included stroke cases regardless of PFO [29]. In these studies, screening for DVT was done by ultrasonography and magnetic resonance venography of the pelvis. Remarkably, the present study used compression ultrasonography only, which resulted in a comparable proportion of patients. This observation leads to the assumption that the use of pelvic magnetic resonance venography, in addition to compression ultrasonography, might have resulted in a higher rate of thrombosis in the population underlying this study. However, the German guideline recommends the complete compression sonography of deep and superficial veins of the whole leg [30], as done in this study. According to these guideline, imaging of pelvic veins is only recommended in case of suspicious ultrasound signals or symptoms indicating pelvic vein thrombosis or during pregnancy, which did not occur in our study group. As explorative studies described DVT rates between 7 and 10.5% in patients with stroke linked to PFO [13], significantly higher DVT rates reported in earlier studies might be related to methodological issues. In detail, one study found DVT in 27% of stroke patients [31], whereby the underlying population was relatively small, with only 37 cases, and those with an etiology classified other than cryptogenic were previously excluded. When focusing on subgroups of stroke and TIA, the present study identified DVT only in patients with stroke. One reason might be that TIA patients exhibited a healthier condition at the time of the cerebrovascular event and thus had a markedly lower risk for DVT, supported by the significantly lower pre-mRS compared to cases with stroke, as seen in this study. Another reason might be that the cohort of patients with TIA in this study was too small to yield cases with DVT.

Regarding parameters associated with DVT in patients with stroke or TIA and concomitant PFO, this study indicated an increased NIHSS at hospital admission, current or past cancer, a history of DVT, immobilization within the last 30 days, coincident pulmonary artery embolism, calf compression pain, and calf circumference difference as features that differ significantly between those with and without sonographic evidence. These observations are comparable with previous investigations in patients other than stroke or TIA. For example, a recent study in unselected patients identified malignancy (OR 2.84, 95%-CI 0.518–15.513), surgery (OR 2.66, 95%-CI 0.411–17.281), and trauma (OR 2.30, 95%-CI 0.452–11.648), the last two are usually accompanied by immobilization, as the three most frequent conditions associated with sonographically confirmed DVT [32]. Further, a study in patients with neurological diseases other than cerebrovascular events found malignant conditions associated with a high risk for DVT (OR 11.7, 95%-CI 1.0–301.4) [15].

Concerning laboratory findings, the present study revealed D-dimer, CRP, LA-specific aPTT, protein S, and genetic thrombophilia as parameters that differ significantly between patients with and without DVT. For D-dimer, comparable observations were made in a study including neurological patients other than cerebrovascular events (OR 5.7, 95%CI 2.1–16.7) [15], in two studies with stroke patients at the time of rehabilitation (OR 1.446, 95%-CI 1.130–1.849 [33], OR 2.283, 95%-CI 1.374–3.868 [34]), and in one study with stroke patients in the earlier stage (1.05, 95%-CI 1.00-1.09 per 1-μg/mL increase) [35]. For CRP, the present study revealed significantly higher CRP serum levels in patients with DVT compared to those without evidence of DVT. This is in good accordance with a previous survey comprising patients with stroke regardless of PFO, while cases with evidence for DVT had an increased CRP compared to those without [29].

Among established scores in the field of DVT and PFO, this study identified the Wells score as significantly increased in patients with DVT compared to those without, with a remarkable risk increase of about 400% for a one-point increase in the score. Even though it did not have such a strong association, the Wells score was also increased in cases with DVT in an earlier study, including 133 stroke patients [35]. Considering parameters identified in the present and previous studies, the finding that the Wells score might help to identify stroke patients with an increased risk of DVT seems plausible as the score combines conditions such as immobilization, malignancy, history of DVT, and difference in calf circumference. Collectively, regarding the cohort of patients with stroke or TIA and concomitant PFO, the Wells score appears suitable for clinical practice as a starting point guiding additional examinations, i.e., compression ultrasonography.

This study has some limitations: Due to its retrospective design and the use of routinely obtained data, some patients with PFO may have been missed, because TEE, which is known to be superior to transthoracic echocardiography (TTE) in detecting PFO [36], is typically performed more often in younger than in older patients. Not all patients with PFO had been examined by compression ultrasonography, further limiting the sample size and generalization of the findings. In addition, as the presence of DVT was based on ultrasound of the lower limbs, sites like deep pelvic and more proximally located veins, which might also represent potential sources of embolic events [27], are not considered. Since ultrasonography of the leg veins was usually performed not directly at hospital admission, it cannot be ruled out that some patients may have acquired deep vein thrombosis during their hospital stay. This could play a role, especially in severely affected patients who are subsequently immobilized. Although a remarkable number of patients was screened, the resulting cohort with concomitant PFO and DVT was relatively small and some laboratory parameters were available only in a few patients, limiting some statistical calculations, i.e., concerning diagnoses (stroke vs. TIA), clinical, and laboratory characteristics. For instance, a normal D-dimer was not seen in the group with DVT, which inhibited the calculation of OR and 95%-CI, while for other parameters, the skew in data might have increased ORs and upper limits of 95%-CI drastically. On the other hand, the emerging limitations clearly illustrate the challenges of investigations regarding PFO and DVT in patients with cerebrovascular events.

## Conclusion

This study indicated a DVT rate of 7.8% in patients with cerebral ischemia and concomitant PFO, which needs to be considered when planning the individual diagnostic workup. A few anamnestic, clinical, and laboratory parameters were identified to be associated with an existing DVT. However, a multivariate regression model with stepwise backward selection identified the Wells score as a significant predictor of DVT. The Wells score thus seems to be suitable to guide additional examinations, i.e., compression ultrasonography for screening of DVT in patients with cerebral ischemia and concomitant PFO.

## Data Availability

Data underlying this study will be made available upon reasonable request to the corresponding author.

## Declarations

### Use of AI-assisted technologies

Grammarly (Grammarly Inc., San Francisco, CA, USA) was used to check and improve the manuscript’s language and readability without applying generative AI services.
